# Supplementary material for: Exploring the relationship between trunk flexibility and arterial stiffness measured by pulse wave velocity: A systematic review and meta-analysis
Source: PLoS One. 2024 Dec 20;19(12):e0311611. doi: 10.1371/journal.pone.0311611 (PMC11661619; doi:10.1371/journal.pone.0311611)
Supplement: S1 File — (DOCX) [file pone.0311611.s002.docx]

**Supplementary material**

**Table S1**. Search strategy for PubMed.

| 1 trunk flexibility [All Fields]  2 pliability [MeSH Terms]  3 pliability [All Fields]  4 flexibility [All Fields]  5 1 OR 2 OR 3 OR 4  6 arterial stiffness [All Fields]  7 aortic stiffening [All Fields] | 8 arterial stiffening [All Fields]  9 pulse wave velocity [All Fields]  10 PWV [All Fields]  11 baPWV [All Fields]  12 cfPWV [All Fields]  13 6 OR 7 OR 8 OR 9 OR 10 OR 11 OR 12  14 5 AND 13 |
| --- | --- |

**Table S2.** Body mass index, systolic blood pressure and diastolic blood pressure mean values of the included studies.

|  | **Poor trunk flexibility** | | | **High trunk flexibility** | | | |
| --- | --- | --- | --- | --- | --- | --- | --- |
| **Reference** | **BMI (kg/m2)** | **SBP (mmHg)** | **DBP (mmHg)** | **BMI (kg/m2)** | **SBP (mmHg)** | **DBP (mmHg)** |  |
| Gando et al. 2017 | 22.3±2.8 | 117.0±14.0 | 71.0±11.0 | 22.2±2.5 | 116.5±12.5 | 70.5±9.5 |  |
| Komatsu et al. 2017 | Middle-age: 22.4±NA  Older: 22.2±NA | Middle-age: 123.0±17.0  Older: 134.0±18.0 | Middle-age: 74.0±10.0  Older: 75.0±12.0 | Middle-age: 22.5±NA  Older: 22.4±NA | Middle-age: 127.0±20.0  Older: 124.0±13.0 | Middle-age: 78.0±10.0  Older: 72.0±9.0 |  |
| Nishiwaki et al. 2014 | Young: 21.5±0.3  Middle-age: 22.9±0.3  Older: 23.1±0.3 | Young: 119.5±1.0  Middle-age: 127.0±1.5  Older: 136.0±2.0 | Young: 71.0±0.7  Middle-age: 81.0±1.0  Older: 82.5±1.0 | Young: 22.0±0.3  Middle-age: 22.8±0.4  Older: 23.0±0.3 | Young: 120.5±1.0  Middle-age: 128.5±2.0  Older: 135.5±2.0 | Young: 71.0±1.0  Middle-age: 82.5±1.0  Older: 82.5±1.0 |  |
| Yamamoto et al. 2009 | Young: 21.6±NA  Middle-age: 23.7±NA  Older: 22.5±NA | Young: 109.0±1.0  Middle-age: 121.0±1.0  Older: 129.0±2.0 | Young: 62.0±1.0  Middle-age: 72.0±1.0  Older: 74.0±1.0 | Young: 21.0±NA  Middle-age: 23.5±NA  Older: 22.6±NA | Young: 110.0±1.0  Middle-age: 116.0±1.0  Older: 124.0±1.0 | Young: 62.0±1.0  Middle-age: 70.0±1.0  Older: 72.0±1.0 |  |
| Yoo et al. 2022 | 23.3±3.0 | 131.7±14.7 | 72.7±9.4 | 24.3±2.5 | 132.5±17.0 | 74.7±8.9 |  |

| **Reference** | **1** | **2** | **3** | **4** | **5** | **6** | **7** | **8** | **9** | **10** | **11** | **12** | **13** | **14** | **Quality** |
| --- | --- | --- | --- | --- | --- | --- | --- | --- | --- | --- | --- | --- | --- | --- | --- |
| Gando et al. 2017 | Y | Y | Y | Y | NR | Y | N | Y | Y | N | Y | NR | Y | Y | Good |
| Komatsu et al. 2017 | Y | Y | Y | Y | NR | Y | NA | Y | Y | NA | Y | NR | NA | Y | Good |
| Nishiwaki et al. 2014 | Y | Y | N | Y | Y | Y | NA | Y | Y | NA | Y | Y | NA | Y | Good |
| Yamamoto et al. 2009 | Y | Y | Y | Y | NR | Y | NA | Y | Y | NA | Y | NR | NA | N | Fair |
| Yoo et al. 2022 | Y | N | Y | Y | NR | Y | NA | Y | N | NA | Y | NR | NA | Y | Fair |

**Table S3.** Quality assessment with the tool for observational cohort and cross-sectional studies of the National Heart, Lung and Blood Institute.

1. Was the research question or objective in this paper clearly stated?; 2. Was the study population clearly specified and defined?; 3. Was the participation rate of eligible persons at least 50%?; 4. Were all the subjects selected or recruited from the same or similar populations (including the same time period)? Were inclusion and exclusion criteria for being in the study prespecified and applied uniformly to all participants?; 5. Was a sample size justification, power description, or variance and effect estimates provided?; 6. For the analyses in this paper, were the exposure(s) of interest measured prior to the outcome(s) being measured?; 7. Was the timeframe sufficient so that one could reasonably expect to see an association between exposure and outcome if it existed?; 8. For exposures that can vary in amount or level, did the study examine different levels of the exposure as related to the outcome (e.g., categories of exposure, or exposure measured as continuous variable)?; 9. Were the exposure measures (independent variables) clearly defined, valid, reliable, and implemented consistently across all study participants?; 10. Was the exposure(s) assessed more than once over time?; 11. Were the outcome measures (dependent variables) clearly defined, valid, reliable, and implemented consistently across all study participants?; 12. Were the outcome assessors blinded to the exposure status of participants?; 13. Was loss to follow-up after baseline 20% or less?; 14. Were key potential confounding variables measured and adjusted statistically for their impact on the relationship between exposure(s) and outcome(s)?; N: no; NA: not applicable; NR: not reported; Y: yes.

**Figure S1**. Forest plot for pooled mean values of the sit-and-reach test for trunk flexibility.

**Figure S2.** Forest plot for pooled mean values of baPWv.

**Figure S3.** Forest plot for pooled mean values of cfPWv.

**Figure S4.** Meta-regresion for mean age as continuous variable and pooled standardized mean differences of PWv.

**Figure S5.** Meta-regresion for body mass index as continuous variable and pooled standardized mean differences of PWv.

**Figure S6.** Meta-regresion for systolic blood pressure as continuous variable and pooled standardized mean differences of PWv.

**Figure S7.** Meta-regresion for diastolic blood pressure as continuous variable and pooled standardized mean differences of PWv.

**Figure S8.** Publication bias for pooled standardized mean differences of PWv.
