# Supplementary material for: Exploring the relationship between trunk flexibility and arterial stiffness measured by pulse wave velocity: A systematic review and meta-analysis
Source: PLoS One. 2024 Dec 20;19(12):e0311611. doi: 10.1371/journal.pone.0311611 (PMC11661619; doi:10.1371/journal.pone.0311611)
Supplement: S2 File — (DOCX) [file pone.0311611.s003.docx]

**Numbered Table of All Studies Identified in the Literature Search, Including Those Excluded from the Analyses:**

|  | Reference | Included/Excluded | Reason of exclusion | Title | DOI |
| --- | --- | --- | --- | --- | --- |
| 1 | Tseng, H.-J. et al., 2013 | Excluded | Irrelevant based on title and abstract review | Flexible PZT Thin Film Tactile Sensor for Biomedical Monitoring | 10.3390/s130505478 |
| 2 | Kruse, N.T. et al., 2020 | Excluded | Irrelevant based on title and abstract review | Comment on: "The Case for Retiring Flexibility as a Major Component of Physical Fitness" | 10.1007/s40279-020-01291-y |
| 3 | Unknow author, 2014 | Excluded | Irrelevant based on title and abstract review | 4th International Conference on Human Performance Development through Strength and Conditioning, NSCA 2014 | No DOI |
| 4 | Badji, A. et al., 2020 | Excluded | Irrelevant based on title and abstract review | A Cross-Sectional Study on the Impact of Arterial Stiffness on the Corpus Callosum, a Key White Matter Tract Implicated in Alzheimer's Disease | 10.3233/JAD-200668 |
| 5 | Peng, Y. et al, 2021 | Excluded | Not outcome of interest | A Flexible Pressure Sensor with Ink Printed Porous Graphene for Continuous Cardiovascular Status Monitoring | 10.3390/s2102048 |
| 6 | Lee, C. et al., 2020 | Excluded | Irrelevant based on title and abstract review | A literature review of the effects of self-myofascial release with a foam roller on human fascial system and cardiovascular function | 10.15857/ksep.2020.29.4.329 |
| 7 | Hiramasu, K. et al, 2004 | Excluded | Irrelevant based on title and abstract review | A novel Arterial Stiffness Index (ASI) as a marker of arteriosclerosis | No DOI |
| 8 | Morgan, K.G., 2019 | Excluded | Irrelevant based on title and abstract review | Actin and focal adhesion remodeling as therapeutic targets in cardiovascular disease | No DOI |
| 9 | Inami, T. et al., 2015 | Excluded | Irrelevant based on title and abstract review | Acute Changes in Peripheral Vascular Tonus and Systemic Circulation during Static Stretching | 10.1080/15438627.2015.1005296 |
| 10 | Yamato, Y. et al., 2016 | Excluded | Irrelevant based on title and abstract review | Acute Effect of Static Stretching Exercise on Arterial Stiffness in Healthy Young Adults | 10.1097/PHM.0000000000000498 |
| 11 | Lee, C. et al., 2021 | Excluded | Irrelevant based on title and abstract review | Acute Effects of Foam Rolling Exercises on Arterial Stiffness, Flexibility and Autonomic Nervous System Function in Young and Middle-Aged Women | 10.15857/ksep.2021.00465 |
| 12 | Chen, C.-H. et al., 2022 | Excluded | Irrelevant based on title and abstract review | Acute Effects of Static Stretching Combined with Vibration and Nonvibration Foam Rolling on the Cardiovascular Responses and Functional Fitness of Older Women with Prehypertension | 10.3390/biology11071025 |
| 13 | Okamoto, T. et al., 2014 | Excluded | Irrelevant based on title and abstract review | Acute effects of self-myofascial release using a foam roller on arterial function | 10.1519/JSC.0b013e31829480f5 |
| 14 | Yang, W.-C. et al., 2021 | Excluded | Irrelevant based on title and abstract review | Acute effects of vibration foam rolling with light and moderate pressure on blood pressure and senior fitness test in older women | 10.3390/ijerph182111186 |
| 15 | Fetter, C. et al., 2020 | Excluded | Irrelevant based on title and abstract review | Additional Improvement of Respiratory Technique on Vascular Function in Hypertensive Postmenopausal Women Following Yoga or Stretching Video Classes: The YOGINI Study | 10.3389/fphys.2020.00898 |
| 16 | Lee, C.K. et al., 2023 | Excluded | Irrelevant based on title and abstract review | Adlay Consumption Combined with Suspension Training Improves Blood Lipids and Pulse Wave Velocity in Middle-Aged Women | 10.3390/healthcare11101426 |
| 17 | Kim, S.-W. et al, 2015 | Excluded | Irrelevant based on title and abstract review | Age-related changes on body composition, functional fitness and arterial compliance in elderly women | No DOI |
| 18 | Cesar, A.M.P., 2017 | Excluded | Irrelevant based on title and abstract review | Aging, Arterial Stiffness and Physical Fitness : Role of Physical Activity and Exercise | No DOI |
| 19 | Whytock, K.L., 208 | Excluded | Irrelevant based on title and abstract review | An Exploration into the Proteins that Regulate Skeletal Muscle Lipid Metabolism | No DOI |
| 20 | Mcgarry, M. et al., 2016 | Excluded | Not population of interest | An inverse approach to determining spatially varying arterial compliance using ultrasound imaging | 10.1088/0031-9155/61/15/5486 |
| 21 | Pasurka, M. et al., 2020 | Excluded | Irrelevant based on title and abstract review | Ankle flossing alters periarticular stiffness and arterial blood flow in asymptomatic athletes | 10.23736/S0022-4707.20.10992-7 |
| 22 | Zhang, R., 2010 | Excluded | Irrelevant based on title and abstract review | Arterial Aging, Brain Perfusion & Exercise: Impact on Brain Structure & Function. | No DOI |
| 23 | Miles, S.C. et al, 2013 | Excluded | Irrelevant based on title and abstract review | Arterial Blood Pressure and Cardiovascular Responses to Yoga Practice | No DOI |
| 24 | Cilhoroz, B.T. et al., 2023 | Excluded | Not outcome of interest | Arterial stiffness and augmentation index are associated with balance function in young adults | 10.1007/s00421-022-05116-w |
| 25 | Huang, Y. et al., 2023 | Excluded | Not outcome of interest | Arteriosclerosis Assessment Based on Single-Point Fingertip Pulse Monitoring Using a Wearable Iontronic Sensor | 10.1002/adhm.202301838 |
| 26 | Bianchini, E. et al., 2008 | Excluded | Irrelevant based on title and abstract review | Assessment of Cardiovascular Risk Markers from Ultrasound Images: System Reproducibility | 10.1109/CIC.2008.4748988 |
| 27 | Hajdusianek, W. et al., 2022 | Excluded | Irrelevant based on title and abstract review | Assessment of aortic stiffness in computed tomography – methodology of radiological examination from 2000 to 2020 | 10.5114/pjr.2022.121556 |
| 28 | Takemura, M. et al., 2015 | Excluded | Irrelevant based on title and abstract review | Association Between Arterial Stiffness And The Shoulder Flexibility. | 10.1249/01.mss.0000476730.42233.dd |
| 29 | Lee, S.S. et al., 2006 | Excluded | Irrelevant based on title and abstract review | Association between arterial stiffness and the deformability of red blood cells (RBCs) | No DOI |
| 30 | Nishitani, N. et al., 2023 | Excluded | Not outcome of interest | Association between trunk flexibility and renal flow pulsatility in middle-aged and older adults | 10.1016/j.exger.2022.112060 |
| 31 | Suwa, M. et al., 2018 | Excluded | Irrelevant based on title and abstract review | Association of body flexibility and carotid atherosclerosis in Japanese middle-aged men: a cross-sectional study | 10.1136/bmjopen-2017-019370 |
| 32 | Lee, Y.H. et al., 2014 | Excluded | Irrelevant based on title and abstract review | Associations of arterial stiffness and cognitive function with physical fitness in patients with chronic stroke | 10.2340/16501977-1790 |
| 33 | Gando, Y. et al., 2021 | Excluded | Irrelevant based on title and abstract review | Body flexibility and incident hypertension: The Niigata wellness study | 10.1111/sms.13867 |
| 34 | Charkoudian, N. et al., 2020 | Excluded | Irrelevant based on title and abstract review | Bursting with flexibility: responsiveness of sympathetic burst size in older women and men | 10.1113/JP280228 |
| 35 | Eldehni,M.T. et al, 2013 | Excluded | Irrelevant based on title and abstract review | BRAIN WHITE MATTER MICROSTRUCTURE USING DIFFUSION TENSOR IMAGING AND COGNITIVE IMPAIRMENT IN HAEMODIALYSIS PATIENTS | No DOI |
| 36 | Thomas, Ewan et al., 2021 | Excluded | Irrelevant based on title and abstract review | Cardiovascular Responses to Muscle Stretching: A Systematic Review and Meta-analysis | 10.1055/a-1312-7131 |
| 37 | Van Schinkel, L.D. et al., 2015 | Excluded | Not population of interest | Cardiovascular flexibility in middle-aged overweight South Asians vs. white Caucasians: response to short-term caloric restriction | 10.1016/j.numecd.2014.12.007 |
| 38 | Lee, Y. et al., 2024 | Excluded | Irrelevant based on title and abstract review | Cardiovascular health monitoring using multiple conformal photoplethysmography devices | 10.1117/12.3010324 |
| 39 | Hou, Y. et al., 2015 | Excluded | Irrelevant based on title and abstract review | Carotid Arterial Stiffness in Patients with Congenital Heart Disease-Related Pulmonary Hypertension Assessed with Radio Frequency Data Technique | 10.1111/echo.12925 |
| 40 | Komatsu, M. et al., 2017 | Included | - | Central blood pressure is associated with trunk flexibility in older adults | 10.1016/j.artres.2017.07.002 |
| 41 | Dobrzeniecki, M. et al., 2018 | Excluded | Irrelevant based on title and abstract review | Cerebral arterial compliance in traumatic brain injury | 10.1007/978-3-319-65798-1_5 |
| 42 | Yamada, E. et al., 2022 | Excluded | Irrelevant based on title and abstract review | Compliance of Static Stretching and the Effect on Blood Pressure and Arteriosclerosis Index in Hypertensive Patients | 10.3390/clinpract12030036 |
| 43 | Gao, P. et al., 2023 | Excluded | Irrelevant based on title and abstract review | Cross-sectional and longitudinal associations between body flexibility and sarcopenia | 10.1002/jcsm.13157 |
| 44 | Ganse, B. et al., 2021 | Excluded | Irrelevant based on title and abstract review | Current Insights in the Age-related Decline in Sports Performance of the Older Athlete | 10.1055/a-1480-7730 |
| 45 | Wang, S. et al., 2022 | Excluded | Not outcome of interest | Development of Pressure Sensor Based Wearable Pulse Detection Device for Radial Pulse Monitoring | 10.3390/mi13101699 |
| 46 | Kim, S. et al., 2008 | Excluded | Irrelevant based on title and abstract review | Development of a Wearable System Module for Monitoring Physical and Mental Workload | 10.1089/tmj.2008.0019 |
| 47 | Duarte, A.F.L., 2020 | Excluded | Irrelevant based on title and abstract review | Efeitos Agudos da Aplicação de Técnicas de Facilitação Proprioceptiva Neuromuscular na Rigidez Arterial em Indivíduos com Doença das Artérias Coronárias | No DOI |
| 48 | Baek, S.H., 2012 | Excluded | Irrelevant based on title and abstract review | Effect of % body fat, physical fitness on impact on cardiovascular risk index in female middle-school students | No DOI |
| 49 | Sadipour, M. et al., 2020 | Excluded | Irrelevant based on title and abstract review | Effect of Aortic Wall Deformation with Healthy and Calcified Annulus on Hemodynamic Performance of Implanted On-X Valve | 10.1007/s13239-019-00453-y |
| 50 | JoWooyeon et al, 2022 | Excluded | Irrelevant based on title and abstract review | Effect of Hatha Yoga on Physical Fitness and Arterial Stiffness in Postmenopausal Women | No DOI |
| 51 | Logan, J.G, 2017 | Excluded | Irrelevant based on title and abstract review | Effect of Static Stretching Exercise on Aortic Pulse Wave Velocity | No DOI |
| 52 | Jeong-min, P. et al., 2020 | Excluded | Irrelevant based on title and abstract review | Effect of Weight Bearing Exercise on Health Related Fitness and Vascular Compliance in Middle Aged Women | 10.51979/KSSLS.2020.01.79.421 |
| 53 | Marciniak, M., 2011 | Excluded | Irrelevant based on title and abstract review | Effect of dietary sodium and potassium on heart, small and large vessel properties | No DOI |
| 54 | Sun, S.H., 2011 | Excluded | Irrelevant based on title and abstract review | Effect of fish oil on aortic elasticity in spontaneously hypertensive rats under medium-and long-term high-fat diet | No DOI |
| 55 | Kalichman, L. et al., 2017 | Excluded | Irrelevant based on title and abstract review | Effect of self-myofascial release on myofascial pain, muscle flexibility, and strength: A narrative review | 10.1016/j.jbmt.2016.11.006 |
| 56 | Yongheelee, et al, 2013 | Excluded | Irrelevant based on title and abstract review | Effect of stretching exercise on arterial stiffness in patients with chronic hemiparesis. | No DOI |
| 57 | Ikegami, H. et al., 1983 | Excluded | Irrelevant based on title and abstract review | Effects Of Physical Training On Body Composition, Respiro-Circulatory Functions, Blood Constituents, And Physical Abilities. Part 1 : Men Aged 30 Years | 10.7600/jspfsm1949.32.302 |
| 58 | Lee, H. et al., 2016 | Excluded | Irrelevant based on title and abstract review | Effects of 8-week Pilates exercise program on menopausal symptoms and lumbar strength and flexibility in postmenopausal women | 10.12965/jer.1632630.315 |
| 59 | Jee, H. et al., 2012 | Excluded | Irrelevant based on title and abstract review | Effects of Abdominal Obesity and Health Related Physical Fitness in Arterial Stiffness of Korean Males | 10.5763/kjsm.2012.30.1.16 |
| 60 | Wong, Alexei et al., 2021 | Excluded | Irrelevant based on title and abstract review | Effects of Acute Stretching Exercise and Training on Heart Rate Variability: A Review | 10.1519/JSC.0000000000003084 |
| 61 | Lee, Y.H. et al., 2015 | Excluded | Irrelevant based on title and abstract review | Effects of Combined Aerobic and Resistance Exercise on Central Arterial Stiffness and Gait Velocity in Patients with Chronic Poststroke Hemiparesis | 10.1097/PHM.0000000000000233 |
| 62 | Sunksom, D. et al, 2017 | Excluded | Irrelevant based on title and abstract review | Effects of Hybrid Exercise Program on Subclinical Vascular Markers in Young Patients with Permanent Lower-Limb Disability | No DOI |
| 63 | Smith, M.F. et al., 2017 | Excluded | Irrelevant based on title and abstract review | Effects of Resistance Band Exercise on Vascular Activity and Fitness in Older Adults | 10.1055/s-0042-121261 |
| 64 | Ko, H.-H., 2022 | Excluded | Irrelevant based on title and abstract review | Effects of Six-week Tai Chi Intervention on Blood Pressure, Brachial-ankle Pulse Wave Velocity and Functional Fitness in Middle-aged and Elderly Hypertensive Patients | No DOI |
| 65 | Logan, J.G. et al., 2018 | Excluded | Not population of interest | Effects of Static Stretching Exercise on Lumbar Flexibility and Central Arterial Stiffness | 10.1097/JCN.0000000000000460 |
| 66 | Choi, J.-H. et al., 2023 | Excluded | Irrelevant based on title and abstract review | Effects of a Mobile-Health Exercise Intervention on Body Composition, Vascular Function, and Autonomic Nervous System Function in Obese Women: A Randomized Controlled Trial | 10.2147/JMDH.S406905 |
| 67 | Ikebe, H. et al., 2023 | Excluded | Irrelevant based on title and abstract review | Effects of acute cervical stretching on arterial wall elastic properties | 10.3389/fphys.2023.1198152 |
| 68 | Lee, Y.H. et al., 2015 | Excluded | Irrelevant based on title and abstract review | Effects of combined aerobic and resistance exercise on central arterial stiffness and gait velocity in patients with chronic poststroke hemiparesis | 10.1097/PHM.0000000000000233 |
| 69 | Wang, S.J. et al., 2015 | Excluded | Irrelevant based on title and abstract review | Effects of estrogen intervention on the biomechanical characteristics of serum SOD, MDA, and middle cerebral artery in aged female rats | 10.12891/ceog1756.2015 |
| 70 | Ferreira, L. et al., 2024 | Excluded | Irrelevant based on title and abstract review | Effects of exercise programs on cardiorespiratory fitness and arterial stiffness on postmenopausal women: A systematic review study | 10.1016/j.maturitas.2024.107917 |
| 71 | Beardsley, C. et al., 2015 | Excluded | Irrelevant based on title and abstract review | Effects of self-myofascial release: A systematic review | 10.1016/j.jbmt.2015.08.007 |
| 72 | Ikebe, H. et al., 2022 | Excluded | Irrelevant based on title and abstract review | Effects of trunk stretching using an exercise ball on central arterial stiffness and carotid arterial compliance | 10.1007/s00421-022-04912-8 |
| 73 | Suh, M.R. et al., 2021 | Excluded | Irrelevant based on title and abstract review | Efficacy of Cord Blood Cell Therapy for Hutchinson-Gilford Progeria Syndrome-A Case Report | 10.3390/ijms222212316 |
| 74 | Sveaas, S.H. et al., 2014 | Excluded | Irrelevant based on title and abstract review | Efficacy of High Intensity Exercise on Disease Activity and Cardiovascular Risk in Active Axial Spondyloarthritis: A Randomized Controlled Pilot Study | 10.1371/journal.pone.0108688 |
| 75 | Suh, M.R. et al., 2021 | Excluded | Irrelevant based on title and abstract review | Efficacy of cord blood cell therapy for hutchinson–gilford progeria syndrome—a case report | 10.3390/ijms222212316 |
| 76 | Sveaas, S.H. et al., 2014 | Excluded | Irrelevant based on title and abstract review | Efficacy of high intensity exercise on disease activity and cardiovascular risk in active axial spondyloarthritis: A randomized controlled pilot study | 10.1371/journal.pone.0108688 |
| 77 | Wong, A. et al., 2014 | Excluded | Irrelevant based on title and abstract review | Eight weeks of stretching training reduces aortic wave reflection magnitude and blood pressure in obese postmenopausal women | 10.1038/jhh.2013.98 |
| 78 | Li, J. et al., 2023 | Excluded | Irrelevant based on title and abstract review | Engineering Smart Composite Hydrogels for Wearable Disease Monitoring | 10.1007/s40820-023-01079-5 |
| 79 | Shinno, H. et al., 2017 | Excluded | Not population of interest | Evaluation of a static stretching intervention on vascular endothelial function and arterial stiffness | 10.1080/17461391.2017.1284267 |
| 80 | Mooventhan, A. et al., 2017 | Excluded | Irrelevant based on title and abstract review | Evidence based effects of yoga practice on various health related problems of elderly people: A review | 10.1016/j.jbmt.2017.01.004 |
| 81 | Smith, J.C., 2020 | Excluded | Irrelevant based on title and abstract review | Exercise for Brain Health with Increased Genetic Risk for Alzheimer's Disease | No DOI |
| 82 | Tang, A. et al., 2014 | Excluded | Irrelevant based on title and abstract review | Exercise-induced changes in cardiovascular function after stroke: A randomized controlled trial | 10.1111/ijs.12156 |
| 83 | Chung, J. et al., 2014 | Excluded | Irrelevant based on title and abstract review | Fitness Predicts Arterial Stiffness Of Men In Healthy Adults | 10.1249/01.mss.0000494163.11547.f1 |
| 84 | Chung, J. et al., 2018 | Excluded | Not population of interest | Fitness as a determinant of arterial stiffness in healthy adult men: A cross-sectional study | 10.23736/S0022-4707.17.06767-6 |
| 85 | Martinez, A.H. et al., 2020 | Excluded | Irrelevant based on title and abstract review | Flexibility Modifies the Age-related Arterial Stiffness in Women With Systemic Lupus Erythematosus | 10.1161/circ.142.suppl_3.15867 |
| 86 | Yamamoto, K., 2007 | Excluded | Irrelevant based on title and abstract review | Flexibility and arterial stiffness | No DOI |
| 87 | Tseng, H.-J. et al., 2013 | Excluded | Irrelevant based on title and abstract review | Flexible PZT thin film tactile sensor for biomedical monitoring | 10.3390/s130505478 |
| 88 | Zhang, X., 2022 | Excluded | Irrelevant based on title and abstract review | Flexible Piezoelectric Array for Cardiovascular MonitoringDuring Cardiac Arrest | No DOI |
| 89 | Luo, N., 2017 | Excluded | Irrelevant based on title and abstract review | Flexible Piezoresistive Sensors for Biomedical Applications | No DOI |
| 90 | Seong, H.M. et al, 2017 | Excluded | Irrelevant based on title and abstract review | Floor Exercise improves on Senior Fitness Test, Blood Lipids and Arterial Stiffness in Elderly Women with Metabolic Syndrome | No DOI |
| 91 | Wang, X. et al., 2023 | Excluded | Not outcome of interest | Flexible Sensors Array Based on Frosted Microstructured Ecoflex Film and TPU Nanofibers for Epidermal Pulse Wave Monitoring | 10.3390/s23073717 |
| 92 | Nishiwaki, M. et al., 2015 | Excluded | Irrelevant based on title and abstract review | Four weeks of regular static stretching reduces arterial stiffness in middle-aged men | 10.1186/s40064-015-1337-4 |
| 93 | Bahloul, M.A. et al., 2024 | Excluded | Not outcome of interest | Fractional-Order Modeling of Arterial Compliance in Vascular Aging: A Computational Biomechanical Approach for Investigating Cardiovascular Dynamics | 10.1109/OJEMB.2023.3343083 |
| 94 | Sawicka, D. et al., 2020 | Excluded | Irrelevant based on title and abstract review | Functional and structural changes in aorta of mice divergently selected for basal metabolic rate | 10.1007/s00360-019-01252-6 |
| 95 | Gando, Y. et al., 2017 | Included | - | Greater Progression of Age-Related Aortic Stiffening in Adults with Poor Trunk Flexibility: A 5-Year Longitudinal Study | 10.3389/fphys.2017.00454 |
| 96 | Islami, F. et al., 2021 | Excluded | Irrelevant based on title and abstract review | Gym and home-based combined training in men with primary hypertension: are they equally effective on functional fitness profile, body composition components, and biochemical parameters of hypertension? | 10.1080/10641963.2021.1960365 |
| 97 | Rapant, S. et al., 2019 | Excluded | Irrelevant based on title and abstract review | Hard Water, More Elastic Arteries: A Case Study from Krupina District, Slovakia | 10.3390/ijerph16091521 |
| 98 | Hunter, S.D. et al., 2013 | Excluded | Irrelevant based on title and abstract review | Hatha yoga and vascular function: Results from cross-sectional and interventional studies | 10.1016/j.jbmt.2012.10.009 |
| 99 | Tian, G. et al., 2024 | Excluded | Not outcome of interest | Hierarchical Piezoelectric Composites for Noninvasive Continuous Cardiovascular Monitoring | 10.1002/adma.202313612 |
| 100 | Kim, Y.-H. et al., 2010 | Excluded | Irrelevant based on title and abstract review | Higher Physical Fitness is Related with Lower Arterial Stiffness in Middle and Old Years Ages | 10.1249/01.MSS.0000384769.32168.c8 |
| 101 | Jenkins, N.D.M. et al., 2022 | Excluded | Irrelevant based on title and abstract review | How do Adverse Childhood Experiences get Under the Skin to Promote Cardiovascular Disease? A Focus on Vascular Health | 10.1093/function/zqac032 |
| 102 | Yoo, T.-K. et al., 2022 | Included | - | Impact of Sex on the Association between Flexibility and Arterial Stiffness in Older Adults | 10.3390/medicina58060789 |
| 103 | Park, S.-Y. et al., 2019 | Excluded | Not population of interest | Impacts of aquatic walking on arterial stiffness, exercise tolerance, and physical function in patients with peripheral artery disease: A randomized clinical trial | 10.1152/japplphysiol.00209.2019 |
| 104 | Russell, R.D. et al., 2022 | Excluded | Irrelevant based on title and abstract review | Impaired postprandial skeletal muscle vascular responses to a mixed meal challenge in normoglycaemic people with a parent with type 2 diabetes | 10.1007/s00125-021-05572-7 |
| 105 | Petritz, A. et al., 2021 | Excluded | Irrelevant based on title and abstract review | Imperceptible energy harvesting device and biomedical sensor based on ultraflexible ferroelectric transducers and organic diodes | 10.1038/s41467-021-22663-6 |
| 106 | Yu, Rouhui et al., 2024 | Excluded | Not outcome of interest | In-situ forming ultra-mechanically sensitive materials for high-sensitivity stretchable fiber strain sensors | 10.1093/nsr/nwae158 |
| 107 | Lopes, G. O. et al., 2019 | Excluded | Irrelevant based on title and abstract review | Increased vascular function and superoxide dismutase activity in physically active vs inactive adults living with HIV | 10.1111/sms.13312 |
| 108 | Myers, R. et al., 2022 | Excluded | Irrelevant based on title and abstract review | Inhibition of the Hippo Pathway Prevents Arterial Stiffness and Hypertension in Vascular Smooth Muscle Cell Specific Kdm6a Knockout Mice | 10.1096/fasebj.2022.36.S1.R5124 |
| 109 | Oviedo, G.R. et al., 2020 | Excluded | Irrelevant based on title and abstract review | Intellectual disability, exercise and aging: The IDEA study: Study protocol for a randomized controlled trial | 10.1186/s12889-020-09353-6 |
| 110 | Sun, Y. et al., 2024 | Excluded | Irrelevant based on title and abstract review | Intelligent cardiovascular disease diagnosis system combined piezoelectric nanogenerator based on 2D Bi2O2Se with deep learning technique | 10.1016/j.nanoen.2024.109878 |
| 111 | Berrones, A.J., 2016 | Excluded | Irrelevant based on title and abstract review | Lifestyle contributors to cardiovascular disease risk | No DOI |
| 112 | Park, S. et al., 2023 | Excluded | Not outcome of interest | Interpolation time-optimized aortic pulse wave velocity estimation by 4D flow MRI | 10.1038/s41598-023-43799-z |
| 113 | Tan, C.H. et al., 2017 | Excluded | Irrelevant based on title and abstract review | Mapping cerebral pulse pressure and arterial compliance over the adult lifespan with optical imaging | 10.1371/journal.pone.0171305 |
| 114 | Douris, P.C. et al., 2013 | Excluded | Irrelevant based on title and abstract review | Martial arts training attenuates arterial stiffness in middle aged adults | 10.5812/asjsm.34259 |
| 115 | Hirano, H. et al., 2011 | Excluded | Irrelevant based on title and abstract review | Measurement of arterial viscoelastic properties using a foil-type pressure sensor and a photoplethysmography | 10.1109/ICEEI.2011.6021689 |
| 116 | Liu, X. et al., 2022 | Excluded | Irrelevant based on title and abstract review | Mechanotransduction regulates inflammation responses of epicardial adipocytes in cardiovascular diseases | 10.3389/fendo.2022.1080383 |
| 117 | Qiao, Z. et al., 2022 | Excluded | Irrelevant based on title and abstract review | Microphysical Characteristics of Cold Cloud Catalytic Snowfall Processes in the Source Region of the Yellow River in Winter | 10.16058/j.issn.1005-0930.2022.05.006 |
| 118 | Zhang, R. et al, 2011 | Excluded | Irrelevant based on title and abstract review | Mild Cognitive Impairment: Cerebrovascular Dysfunction and Exercise Training | No DOI |
| 119 | Ives, S.J. et al., 2017 | Excluded | Irrelevant based on title and abstract review | Multi-modal exercise training and protein -pacing enhances physical performance adaptations independent of growth hormone and BDNF but may be dependent on IGF-1 in exercise-trained men | 10.1016/j.ghir.2016.10.002 |
| 120 | Poli, L. et al., 2024 | Excluded | Irrelevant based on title and abstract review | Multicomponent versus aerobic exercise intervention: Effects on hemodynamic, physical fitness and quality of life in adult and elderly cardiovascular disease patients: A randomized controlled study | 10.1016/j.heliyon.2024.e36200 |
| 121 | Macpherson, H. et al., 2019 | Excluded | Irrelevant based on title and abstract review | Multifaceted intervention to enhance cognition in older people at risk of cognitive decline: Study protocol for the Protein Omega-3 and Vitamin D Exercise Research (PONDER) study | 10.1136/bmjopen-2018-024145 |
| 122 | Trasande, L., 2022 | Excluded | Irrelevant based on title and abstract review | NYU Pediatric Obesity, Metabolism and Kidney Cohort Center | No DOI |
| 123 | Bowie, D.C. et al., 2024 | Excluded | Irrelevant based on title and abstract review | Neurovascular mechanisms of cognitive aging: Sex-related differences in the average progression of arteriosclerosis, white matter atrophy, and cognitive decline | 10.1016/j.nbd.2024.106653 |
| 124 | Mankowski, R.T., 2020 | Excluded | Irrelevant based on title and abstract review | Nicotinamide riboside as an Enhancer of Exercise Therapy in hypertensive older adults: The NEET Trial | No DOI |
| 125 | Hirano, H. et al., 2011 | Excluded | Irrelevant based on title and abstract review | Noninvasive estimation of arterial viscoelastic indices using a foil-type flexible pressure sensor and a photoplethysmogram | 10.15676/ijeei.2011.3.4.9 |
| 126 | Hart, N R et al., 2012 | Excluded | Irrelevant based on title and abstract review | OS091. The origin of preeclampsia in diet-induced maternal endothelial stiffness. | 10.1016/j.preghy.2012.04.092 |
| 127 | Coles, C.D. et al, 2021 | Excluded | Irrelevant based on title and abstract review | PRENATAL ALCOHOL EXPOSURE AND COGNITIVE OUTCOMES IN MIDLIFE: LONG-TERM EFFECTS IN TWO LONGITUDINAL COHORTS | No DOI |
| 128 | Li, Y. et al, 2009 | Excluded | Irrelevant based on title and abstract review | Perindopril, amlodipine and telmisartan improve arterial stiffness in patients with hypertension | No DOI |
| 129 | Soriano-Maldonado, A. et al., 2020 | Excluded | Irrelevant based on title and abstract review | Physical Exercise following bariatric surgery in women with Morbid obesity Study protocol clinical trial (SPIRIT compliant) | 10.1097/MD.0000000000019427 |
| 130 | Sola-Rodríguez, S. et al., 2021 | Excluded | Irrelevant based on title and abstract review | Physical Fitness Attenuates the Impact of Higher Body Mass and Adiposity on Inflammation in Women With Systemic Lupus Erythematosus | 10.3389/fimmu.2021.729672 |
| 131 | Gando, Y., 2016 | Excluded | Irrelevant based on title and abstract review | Poor Trunk Flexibility is Associated With Greater Progression of Age-related Arterial Stiffening: A 5-year Longitudinal Study | No DOI |
| 132 | Yi, Z. et al., 2022 | Excluded | Not outcome of interest | Piezoelectric Dynamics of Arterial Pulse for Wearable Continuous Blood Pressure Monitoring | 10.1002/adma.202110291 |
| 133 | Ogawa, N. et al., 2020 | Excluded | Not outcome of interest | Poor Walking Speed Is Associated With Higher Segment-Specific Arterial Stiffness in Older Adult Japanese Community Dwellers: A Cross-Sectional Study | 10.3389/fphys.2020.587215 |
| 134 | Yamamoto, K. et al., 2009 | Included | - | Poor trunk flexibility is associated with arterial stiffening | 10.1152/ajpheart.00061.2009 |
| 135 | Davy, K.P., 2014 | Excluded | Irrelevant based on title and abstract review | Prebiotics, Gut Microbiota, and Cardiometabolic Health | No DOI |
| 136 | Ruiz, J.R. et al., 2009 | Excluded | Irrelevant based on title and abstract review | Predictive validity of health-related fitness in youth: A systematic review | 10.1136/bjsm.2008.056499 |
| 137 | Williams, A.D. et al., 2013 | Excluded | Irrelevant based on title and abstract review | Progressive resistance training might improve vascular function in older women but not in older men | 10.1016/j.jsams.2012.05.001 |
| 138 | Huotari, M. et al., 2013 | Excluded | Irrelevant based on title and abstract review | Pulse waveforms are an indicator of the condition of vascular system | 10.1007/978-3-642-29305-4_138 |
| 139 | Zahedi, E. et al., 2013 | Excluded | Irrelevant based on title and abstract review | Quick deployment of open-source hardware and software for a dual-channel biosignal recorder | 10.1109/ICSIMA.2013.6717933 |
| 140 | Rao, U., 2023 | Excluded | Irrelevant based on title and abstract review | Racial/Ethnic Influences on Early Vascular Aging and Cardiac Strain: Role of Cumulative Stress, Inflammatory and Metabolic Burden | No DOI |
| 141 | Ikebe, H. et al., 2022 | Excluded | Irrelevant based on title and abstract review | Regular exercise ball training reduces arterial stiffness in sedentary middle-aged males. | 10.1589/jpts.34.386 |
| 142 | Satiroglu, O. et al., 2012 | Excluded | Irrelevant based on title and abstract review | Relation between aortic stiffness and extension of coronary artery disease | 10.3906/sag-1101-1424 |
| 143 | Ma, Y. et al., 2018 | Excluded | Not population of interest | Relation between blood pressure and pulse wave velocity for human arteries | 10.1073/pnas.1814392115 |
| 144 | Ko, D.H. et al., 2022 | Excluded | Irrelevant based on title and abstract review | Relationship between cardiovascular disease risk factors, health behavior and physical fitness according to visceral fat in older men | 10.31083/j.jomh1806128 |
| 145 | Park, S.-H. et al, 2012 | Excluded | Irrelevant based on title and abstract review | Relationship between flexibility and arterial stiffness in patients with stroke. | No DOI |
| 146 | Turzyniecka, M. J., 2011 | Excluded | Irrelevant based on title and abstract review | Relationships between cardio-metabolic risk factors in central obesity and the effects of high dose statin treatment | No DOI |
| 147 | Yamamoto, K., 2009 | Excluded | Irrelevant based on title and abstract review | Relationships between flexibility, arterial stiffening and the sympathetic nervous system | No DOI |
| 148 | Zheng, P. et al., 2024 | Excluded | Irrelevant based on title and abstract review | Remotely-delivered exercise training program for improving physical and cognitive functions among older adults with multiple sclerosis: Protocol for an NIH stage-I randomized controlled trial | 10.1016/j.cct.2024.107636 |
| 149 | Vargas-Delgado, A.P. et al., 2023 | Excluded | Not outcome of interest | Renal and Cardiovascular Metabolic Impact Caused by Ketogenesis of the SGLT2 Inhibitors | 10.3390/ijms24044144 |
| 150 | Wang, X. et al., 2013 | Excluded | Irrelevant based on title and abstract review | Research on biomechanics properties for balloon-expandable intracoronary stents | 10.3969/j.issn.0258-8021.2013.02.012 |
| 151 | Kumagai, H. et al., 2019 | Excluded | Not population of interest | Role of High Physical Fitness in Deterioration of Male Sexual Function in Japanese Adult Men | 10.1177/1557988319849171 |
| 152 | Nishiwaki, M. et al., 2014 | Included | - | Sex Differences in Flexibility-Arterial Stiffness Relationship and Its Application for Diagnosis of Arterial Stiffening: A Cross-Sectional Observational Study | 10.1371/journal.pone.0113646 |
| 153 | Winter, P. et al., 2021 | Excluded | Not outcome of interest | Simultaneous measurements of 3D wall shear stress and pulse wave velocity in the murine aortic arch | 10.1186/s12968-021-00725-4 |
| 154 | Hashikata, T. et al., 2019 | Excluded | Irrelevant based on title and abstract review | Stent recoil in overlapping stent 18 years after wiktor stent implantation | 10.1536/ihj.18-261 |
| 155 | Zia, A.W. et al., 2022 | Excluded | Irrelevant based on title and abstract review | Structural design and mechanical performance of composite vascular grafts | 10.1007/s42242-022-00201-7 |
| 156 | Jung, H.C. et al, 2016 | Excluded | Irrelevant based on title and abstract review | Taekwondo training improves CVD risk factors in obese male adolescents | No DOI |
| 157 | Luo, N. et al., 2018 | Excluded | Irrelevant based on title and abstract review | Textile-Enabled Highly Reproducible Flexible Pressure Sensors for Cardiovascular Monitoring | 10.1002/admt.201700222 |
| 158 | Aminuddin, A. et al., 2021 | Excluded | Irrelevant based on title and abstract review | The Association Between Arterial Stiffness and Muscle Indices Among Healthy Subjects and Subjects With Cardiovascular Risk Factors: An Evidence-Based Review | 10.3389/fphys.2021.742338 |
| 159 | Young, L.J. et al, 2012 | Excluded | Irrelevant based on title and abstract review | The Effect of 16 weeks of Exercise Program on Health-Related Physical Fitness and Arterial Stiffness in Elderly with Dementia | No DOI |
| 160 | Hunter, S.D. et al., 2013 | Excluded | Irrelevant based on title and abstract review | The Effect of Bikram Yoga on Arterial Stiffness in Young and Older Adults | 10.1089/acm.2012.0709 |
| 161 | SongChae-Hoon et al, 2016 | Excluded | Irrelevant based on title and abstract review | The Effect of Combined Exercise on Daily Living Fitness and Arterial Stiffness in the obese Elderly Woman | No DOI |
| 162 | Konstantaki, M. et al., 2015 | Excluded | Irrelevant based on title and abstract review | The Effects Of A Pilates Intervention On Arterial Stiffness And Trunk Flexibility | 10.1249/01.mss.0000477709.39123.82 |
| 163 | West, K. et al., 2024 | Excluded | Irrelevant based on title and abstract review | The acute effect of passively assisted trunk stretching on central arterial stiffness and blood pressure in middle-aged to older adults | 10.1007/s00421-023-05389-9 |
| 164 | Hashizume, Y. et al., 2024 | Excluded | Irrelevant based on title and abstract review | The beneficial effects of monoglucosyl hesperidin and monoglucosyl rutin on vascular flexibility: A randomized, placebo-controlled, double-blind, parallel-group study | 10.31989/ffhd.v14i5.1319 |
| 165 | Mitchell, C.M. et al., 2015 | Excluded | Irrelevant based on title and abstract review | The effect of prebiotic supplementation with inulin on cardiometabolic health: Rationale, design, and methods of a controlled feeding efficacy trial in adults at risk of type 2 diabetes | 10.1016/j.cct.2015.10.012 |
| 166 | Song, J. et al, 2022 | Excluded | Irrelevant based on title and abstract review | The Effect of Modified Tai Chi Exercises on the Physical Function and Quality of Life in Elderly Women With Knee Osteoarthritis | 10.3389/fnagi.2022.860762 |
| 167 | Verveniotis, A. et al., 2018 | Excluded | Irrelevant based on title and abstract review | The impact of omega 3 fatty acids in atherosclerosis and arterial stiffness: An overview of their actions | 10.2174/1381612824666180321095022 |
| 168 | Hajjaji, R. et al., 2012 | Excluded | Irrelevant based on title and abstract review | The influence of textile vascular prosthesis crimping on graft longitudinal elasticity and flexibility | 10.1016/j.jmbbm.2012.06.017 |
| 169 | Kishabongo, A.S. et al., 2016 | Excluded | Irrelevant based on title and abstract review | The presence of fructosamine in human aortic valves is associated with valve stiffness | 10.1136/jclinpath-2015-203409 |
| 170 | Bovolini, A. Et al, 2022 | Excluded | Irrelevant based on title and abstract review | Impact of Exercise on Vascular Function in Middle-Aged and Older Adults: A Scoping Review | 10.3390/sports10120208 |
| 171 | Alastruey-Arimon, J. Et al, 2013 | Excluded | Irrelevant based on title and abstract review | Uncovering Contributors to Hypertension through Experimental and Computational Simulation (CHECS) | No DOI |
| 172 | Imbert, J.-P. et al., 2022 | Excluded | Not outcome of interest | Vascular Function Recovery Following Saturation Diving | 10.3390/medicina58101476 |
| 173 | Keller, A., 2029 | Excluded | Irrelevant based on title and abstract review | Vascular Health and Mental Health in Collegiate Female Varsity Athletes, Club Sport Athletes and Recreationally Active Women | No DOI |
| 174 | Sim, H.K., 2020 | Excluded | Irrelevant based on title and abstract review | Wearable Strain Sensors for Measuring Diastolic Blood Pressure | No DOI |
| 175 | El Abbasi, M.K. et al., 2022 | Excluded | Not outcome of interest | Wearable Blood Pressure Sensing Based on Transmission Coefficient Scattering for Microstrip Patch Antennas | 10.3390/s22113996 |
| 176 | Anbuselvam, B. et al., 2024 | Excluded | Not outcome of interest | Wearable biosensors in cardiovascular disease | 10.1016/j.cca.2024.119766 |
| 177 | Meyer, M. et al., 2018 | Excluded | Not population of interest | Web-Based Motor Intervention to Increase Health-Related Physical Fitness in Children With Congenital Heart Disease: A Study Protocol | 10.3389/fped.2018.00224 |
| 178 | Beuchel, C. et al., 2022 | Excluded | Irrelevant based on title and abstract review | Whole Blood Metabolite Profiles Reflect Changes in Energy Metabolism in Heart Failure | 10.3390/metabo12030216 |
| 179 | Whytock, K.L. et al., 2021 | Excluded | Irrelevant based on title and abstract review | Young, healthy males and females present cardiometabolic protection against the detrimental effects of a 7-day high-fat high-calorie diet | 10.1007/s00394-020-02357-3 |
| 180 | Du, L.-g. et al, 2010 | Excluded | Irrelevant based on title and abstract review | [Association between multi-noninvasive indexes and mild coronary stenosis]. | No DOI |
| 181 | Rosset, E. et al, 1996 | Excluded | Irrelevant based on title and abstract review | [Mechanical properties of the arteries. Effects of cryopreservation] | No DOI |
| 182 | Munagala, V.M. et al, 2005 | Excluded | Irrelevant based on title and abstract review | Ventricular structure and function in aged dogs with renal hypertension: a model of experimental diastolic heart failure | 10.1161/01.CIR.0000157183.21404.63 |
| 183 | Tuday, E.C. et al, 2009 | Excluded | Irrelevant based on title and abstract review | Simulated microgravity-induced aortic remodeling | 10.1152/japplphysiol.90777.2008 |
| 184 | Midyett, L.K. et al, 2009 | Excluded | Irrelevant based on title and abstract review | Noninvasive radial artery tonometry augmentation index and urinary albumin/creatinine levels in early adolescents with type 1 diabetes mellitus | 10.1515/jpem.2009.22.6.531 |
| 185 | Urbina, E.M. et al, 2011 | Excluded | Irrelevant based on title and abstract review | Relationship between elevated arterial stiffness and increased left ventricular mass in adolescents and young adults | 10.1016/j.jpeds.2010.12.020 |
| 186 | Saito, M. et al, 2011 | Excluded | Irrelevant based on title and abstract review | One-dimensional model for propagation of a pressure wave in a model of the human arterial network: comparison of theoretical and experimental results | 10.1115/1.4005472 |
| 187 | Li, Y. et al, 2011 | Excluded | Irrelevant based on title and abstract review | Variation of wave speed determined by the PU-loop with proximity to a reflection site | 10.1109/IEMBS.2011.6090032 |
| 188 | Levi-Marpillat, N. et al, 2013 | Excluded | Irrelevant based on title and abstract review | Crucial importance of using a sliding calliper to measure distance for carotid-femoral pulse wave velocity assessment | 10.1097/HJH.0b013e32835e2a2f |
| 189 | Borlotti, A. et al, 2014 | Excluded | Irrelevant based on title and abstract review | Experimental evaluation of local wave speed in the presence of reflected waves | 10.1016/j.jbiomech.2013.10.007 |
| 190 | Desamericq, G. et al, 2015 | Excluded | Irrelevant based on title and abstract review | Carotid-femoral pulse wave velocity is not increased in obesity | 10.1093/ajh/hpu190 |
| 191 | Morris, L. et al, 2016 | Excluded | Irrelevant based on title and abstract review | An Experimental Evaluation of Device/Arterial Wall Compliance Mismatch for Four Stent-Graft Devices and a Multi-layer Flow Modulator Device for the Treatment of Abdominal Aortic Aneurysms | No DOI |
| 192 | Chou, H.C. et al, 2015 | Excluded | Irrelevant based on title and abstract review | Development a polymer-based electronic pulse diagnosis instrument for measuring and analyzing pulse wave velocity | No DOI |
| 193 | Boutry, C.M. et al, 2015 | Excluded | Irrelevant based on title and abstract review | A Sensitive and Biodegradable Pressure Sensor Array for Cardiovascular Monitoring | 10.1002/adma.201502535 |
| 194 | Roberts, P.A. et al, 2015 | Excluded | Irrelevant based on title and abstract review | Real-time aortic pulse wave velocity measurement during exercise stress testing | 10.1186/s12968-015-0191-4 |
| 195 | Hacham, W.S. et al, 2015 | Excluded | Irrelevant based on title and abstract review | Wave speed and reflections proximal to aneurism and stenosis of flexible tubes | 10.1109/EMBC.2015.7318535 |
| 196 | Fu, Y. et al, 2019 | Excluded | Irrelevant based on title and abstract review | A Wearable Multifunctional Pulse Monitor Using Thermosensation-Based Flexible Sensors | 10.1109/TBME.2018.2873754 |
| 197 | Sum, Y. et al, 2018 | Excluded | Irrelevant based on title and abstract review | Wearable Pulse Wave Monitoring System Based on MEMS Sensors | 10.3390/mi9020090 |
| 198 | Chen, J. Et al, 2021 | Excluded | Irrelevant based on title and abstract review | Three-Dimensional Arterial Pulse Signal Acquisition in Time Domain Using Flexible Pressure-Sensor Dense Arrays | 10.3390/mi12050569 |
| 199 | Xu, L. et al, 2022 | Excluded | Irrelevant based on title and abstract review | A Flexible Ultrasound Array for Local Pulse Wave Velocity Monitoring | 10.3390/bios12070479 |
| 200 | Wang, X. Et al, 2022 | Excluded | Irrelevant based on title and abstract review | Artificial Tactile Recognition Enabled by Flexible Low-Voltage Organic Transistors and Low-Power Synaptic Electronics | 10.1021/acsami.2c14625 |
| 201 | Bruns, A. et al, 2024 | Excluded | Irrelevant based on title and abstract review | Plant-based diets and cardiovascular risk factors: a comparison of flexitarians, vegans and omnivores in a cross-sectional study | 10.1186/s40795-024-00839-9 |
| 202 | Patil, S.G. et al, 2024 | Excluded | Irrelevant based on title and abstract review | Effectiveness of yoga on arterial stiffness: A systematic review | 10.1016/j.ctim.2020.102484 |
| 203 | Cinquino, M. et al, 2024 | Excluded | Irrelevant based on title and abstract review | Enhancing cardiovascular health monitoring: Simultaneous multi-artery cardiac markers recording with flexible and bio-compatible AlN piezoelectric sensors | 10.1016/j.bios.2024.116790 |
| 204 | Bargiel, W. et al, 2023 | Excluded | Irrelevant based on title and abstract review | Elastin | No DOI |
| 205 | Huotari, M. Et al, 2013 | Excluded | Irrelevant based on title and abstract review | Pulse waveforms are an indicator of the condition of vascular system | 10.1007/978-3-642-29305-4_138 |
| 206 | Fabiani, M. et al, 2019 | Excluded | Irrelevant based on title and abstract review | Role of arterial stiffness in the decline of cognitive control in aging | No DOI |
| 207 | Tanaka, H. et al, 2015 | Excluded | Irrelevant based on title and abstract review | Effects of Regular Exercise on Arterial Stiffness | No DOI |
| 208 | Fahs, C.A. et al, 2010 | Excluded | Irrelevant based on title and abstract review | Muscular Strength is Inversely Associated with Aortic Stiffness in Young Men | 10.1249/MSS.0b013e3181d8d834 |
| 209 | Lan, Y.S. et al, 2023 | Excluded | Irrelevant based on title and abstract review | Effect of Exercise on Arterial Stiffness in Healthy Young, Middle-Aged and Older Women: A Systematic Review | 10.3390/nu15020308 |
| 210 | Sutton-Tyrrell, K. et al, 2005 | Excluded | Irrelevant based on title and abstract review | Elevated Aortic Pulse Wave Velocity, a Marker of Arterial Stiffness, Predicts Cardiovascular Events in Well-Functioning Older Adults | 10.1161/CIRCULATIONAHA.104.483628 |
| 211 | Boreham, C.A. et al, 2004 | Excluded | Irrelevant based on title and abstract review | Cardiorespiratory Fitness, Physical Activity, and Arterial Stiffness: The Northern Ireland Young Hearts Project | 10.1161/01.HYP.0000144293.40699.9 |
| 212 | Wong, A. et al, | Excluded | Irrelevant based on title and abstract review | The Effects of Stretching Training on Cardiac Autonomic Function in Obese Postmenopausal Women | No DOI |
| 213 | Heffernan, K.S. et al, 2007 | Excluded | Irrelevant based on title and abstract review | Arterial stiffness and baroreflex sensitivity following bouts of aerobic and resistance exercise | 10.1055/s-2006-924290 |
| 214 | Albin, E.E. et al, 2020 | Excluded | Irrelevant based on title and abstract review | Cardiorespiratory Fitness and Muscular Strength on Arterial Stiffness in Older Adults | 10.1249/MSS.0000000000002319 |
| 215 | Hernandez-Martinez, A., et al, 2021 | Excluded | Irrelevant based on title and abstract review | Ideal cardiovascular health in women with systemic lupus erythematosus: Association with arterial stiffness, inflammation, and fitness | 10.1016/j.ijcard.2021.02.040 |
| 216 | Weberruß, H., et al, 2017 | Excluded | Irrelevant based on title and abstract review | Reduced arterial stiffness in very fit boys and girls | 10.1017/S1047951116000226 |
| 217 | Cai, L., et al, 2021 | Excluded | Irrelevant based on title and abstract review | Effects of mind-body practice on arterial stiffness, central hemodynamic parameters and cardiac autonomic function of college students | 10.1016/j.ctcp.2021.101492 |
| 218 | Alhalimi, T.A. et al, 2023 | Excluded | Irrelevant based on title and abstract review | Effects of body positions on arterial stiffness as assessed by pulse wave velocity | 10.1097/HJH.0000000000003418 |
| 219 | Lim, E. et al, 2020 | Excluded | Irrelevant based on title and abstract review | The acute effects of different frequencies of whole-body vibration on arterial stiffness | 10.1080/10641963.2019.1665675 |
| 220 | Otsuki, T. et al, 2008 | Excluded | Irrelevant based on title and abstract review | Arterial stiffness acutely decreases after whole-body vibration in humans | 10.1111/j.1748-1716.2008.01869.x |
| 221 | Sanchez-Gonzalez, M.A. et al, 2011 | Excluded | Irrelevant based on title and abstract review | Creatine supplementation attenuates hemodynamic and arterial stiffness responses following an acute bout of isokinetic exercise | 10.1007/s00421-011-1832-4 |
| 222 | Shimawaki, S., et al, 2015 | Excluded | Irrelevant based on title and abstract review | The effect of measurement position on brachial-ankle pulse wave velocity | No DOi |
| 223 | Takami, T., 2009 | Excluded | Irrelevant based on title and abstract review | Evaluation of arterial stiffness in morning hypertension under high-dose valsartan compared to valsartan plus low-dose diuretic | 10.1038/hr.2009.156 |
| 224 | Sobrinho, A.C.dS. et al, 2021 | Excluded | Irrelevant based on title and abstract review | Effect of Flexibility Training Associated with Multicomponent Training on Posture and Quality of Movement in Physically Inactive Older Women: A Randomized Study | 10.3390/ijerph182010709 |
| 225 | Araújo, C.G. et al, 2024 | Excluded | Irrelevant based on title and abstract review | Reduced Body Flexibility Is Associated With Poor Survival in Middle-Aged Men and Women: A Prospective Cohort Study | 10.1111/sms.14708 |
| 226 | Son, W.-M. et al, 2017 | Excluded | Irrelevant based on title and abstract review | Combined exercise reduces arterial stiffness, blood pressure, and blood markers for cardiovascular risk in postmenopausal women with hypertension | 10.1097/GME.0000000000000765 |
| 227 | Gauthier, C.J. et al, 2015 | Excluded | Irrelevant based on title and abstract review | Hearts and minds: linking vascular rigidity and aerobic fitness with cognitive aging | 10.1016/j.neurobiolaging.2014.08.018 |
| 228 | Mason, J.R. et al, 2022 | Excluded | Irrelevant based on title and abstract review | Arterial Stiffness and Cardiorespiratory Fitness Are Associated With Cognitive Function in Older Adults | 10.1080/08964289.2020.1825921 |
| 229 | Nihei, S. et al, 2023 | Excluded | Irrelevant based on title and abstract review | Arterial stiffness and physical fitness on cognitive function in community-dwelling middle-aged and older adults | 10.1007/s40520-023-02470-3 |
| 230 | Tam, C.H. et al, 2017 | Excluded | Irrelevant based on title and abstract review | Mapping cerebral pulse pressure and arterial compliance over the adult lifespan with optical imaging | 10.1371/journal.pone.0171305 |
| 231 | Tomoto, T. et al, 2021 | Excluded | Irrelevant based on title and abstract review | One-Year Aerobic Exercise Reduced Carotid Arterial Stiffness and Increased Cerebral Blood Flow in Amnestic Mild Cognitive Impairment | 10.3233/JAD-201456 |
| 232 | Demirel, A. et al, 2019 | Excluded | Irrelevant based on title and abstract review | Comparison of vascular arterial stiffness parameters of adolescent wrestlers with healthy subjects: Is heavy training harmful for wrestlers? | 10.3233/BMR-171083 |
| 233 | Valencia Hernandez, C.A., 2019 | Excluded | Irrelevant based on title and abstract review | Arterial stiffness, cardiovascular risk and physical functioning in the Whitehall II study | No DOI |

*Note: Included studies are those mentioned in the systematic review document. No unpublished studies were noted.

**Table of All Data Extracted from Primary Research Sources for the Systematic Review and/or Meta-analysis:**

| **Reference** | **Data Extractors** | **Date of Data Extraction** | **Confirmation of Eligibility** | **Data Extracted** |
| --- | --- | --- | --- | --- |
| Gando et al., 2017 | AS-L, IC-R | 15/03/2024 | Yes | Country, PWV type, Sample size, Age, Flexibility, BMI, SBP, DBP, PWV levels |
| Komatsu et al., 2017 | AS-L, IC-R | 17/03/2024 | Yes | Country, PWV type, Sample size, Age, Flexibility, PWV levels |
| Nishiwaki et al., 2014 | AS-L, IC-R | 18/03/2024 | Yes | Country, PWV type, Sample size, Age, Flexibility, PWV level |
| Yamamoto et al., 2009 | AS-L, IC-R | 19/03/2024 | Yes | Country, PWV type, Sample size, Age, Flexibility, PWV level |
| Yoo et al., 2022 | AS-L, IC-R | 20/03/2024 | Yes | Country, PWV type, Sample size, Age, BMI, SBP, DBP, Flexibility, PWV level |

**Table Showing the Completed Risk of Bias Assessments for Each Study or Outcome:**

| **Reference** | **Domain 1** | **Domain 2** | **Domain 3** | **Domain 4** | **Domain 5** | **Domain 6** | **Domain 7** | **Domain 8** | **Domain 9** | **Domain 10** | **Overall Quality** |
| --- | --- | --- | --- | --- | --- | --- | --- | --- | --- | --- | --- |
| Gando et al., 2017 | Y | Y | Y | Y | NR | Y | N | Y | Y | N | Good |
| Komatsu et al., 2017 | Y | Y | Y | Y | NR | Y | NA | Y | Y | NA | Good |
| Nishiwaki et al., 2014 | Y | Y | N | Y | Y | Y | NA | Y | Y | NA | Good |
| Yamamoto et al., 2009 | Y | Y | Y | Y | NR | Y | NA | Y | Y | NA | Fair |
| Yoo et al., 2022 | Y | N | Y | Y | NR | Y | NA | Y | N | NA | Fair |

*Domains evaluated according to the NIH quality assessment tool. Y: Yes, N: No, NR: Not Reported, NA: Not Applicable.

**Explanation of How Missing Data Were Handled:**

In the studies included in the systematic review and meta-analysis, no critical missing data for the variables of interest were reported.
